# Modeling Analysis of Axonal After Potential at Hippocampal Mossy Fibers

**DOI:** 10.3389/fncel.2019.00210

**Published:** 2019-05-14

**Authors:** Haruyuki Kamiya

**Affiliations:** Department of Neurobiology, Graduate School of Medicine, Hokkaido University, Sapporo, Japan

**Keywords:** axon, action potential, after potential, capacitive discharge, simulation

## Abstract

Action potentials reliably propagate along the axons, and after potential often follows the axonal action potentials. After potential lasts for several tens of millisecond and plays a crucial role in regulating excitability during repetitive firings of the axon. Several mechanisms underlying the generation of after potential have been suggested, including activation of ionotropic autoreceptors, accumulation of K^+^ ions in the surrounding extracellular space, the opening of slow voltage-dependent currents, and capacitive discharge of upstream action potentials passively propagated through axon cable. Among them, capacitive discharge is difficult to examine experimentally, since the quantitative evaluation of a capacitive component requires simultaneous recordings from at least two different sites on the connecting axon. In this study, a series of numerical simulation of the axonal action potential was performed using a proposed model of the hippocampal mossy fiber where morphological as well as electrophysiological data are accumulated. To evaluate the relative contribution of the capacitive discharge in axonal after potential, voltage-dependent Na^+^ current as well as voltage-dependent K^+^ current was omitted from a distal part of mossy fiber axons. Slow depolarization with a similar time course with the recorded after potential in the previous study was left after blockade of Na^+^ and K^+^ currents, suggesting that a capacitive component contributes substantially in axonal after potential following propagating action potentials. On the other hand, it has been shown that experimentally recorded after potential often showed clear voltage-dependency upon changes in the initial membrane potential, obviously deviating from voltage-independent nature of the capacitive component. The simulation revealed that activation of voltage-dependent K^+^ current also contributes to shape a characteristic waveform of axonal after potential and reconstitute similar voltage-dependency with that reported for the after potential recorded from mossy fiber terminals. These findings suggest that the capacitive component reflecting passive propagation of upstream action potential substantially contributes to the slow time course of axonal after potential, although voltage-dependent K^+^ current provided a characteristic voltage dependency of after potential waveform.

## Introduction

The axon carries neuronal information reliably to the presynaptic terminal as a form of the action potential (Debanne et al., 2011). Propagation of action potential along axon has been considered as a highly reliable digital process (Bean, 2007), although the excitability of axons is slightly modulated for a short period after generation of action potential up to tens to hundreds of milliseconds (Gardner-Medwin, 1972; Zucker, 1974). This post-stimulus change in the excitability of axon was mediated, at least in part, by depolarizing after potential which often follows action potential in the axon (Barrett and Barrett, 1982). After potential in axon may be important for temporal integration of neuronal signaling in the brain by affecting short-term plasticity of presynaptic transmitter release (for review, Ohura and Kamiya, 2016). For the candidate cellular mechanisms of axonal after potential, several possibilities have been suggested so far. These include not only passive mechanism reflecting an intrinsic property of axonal membrane (i.e., capacitive discharge of upstream action potentials; Barrett and Barrett, 1982; Borst et al., 1995; David et al., 1995), but also active mechanism due to slow activation of voltage-dependent channels (i.e., persistent-type or resurgent-type Na^+^-channels; Kim et al., 2010; Ohura and Kamiya, 2018b) or extrinsic mechanisms such as K^+^ accumulation (Malenka et al., 1981; Meeks and Mennerick, 2004) or autoreceptor activation (Kamiya et al., 2002; de San Martin et al., 2017). The relative contribution of active and passive mechanisms varies among different type of axons (Barrett and Barrett, 1982; Borst et al., 1995; Kim et al., 2010; Ohura and Kamiya, 2018b), although passive mechanisms due to capacitive discharge commonly underlie axonal depolarizing after potential. Despite functional significance in temporal integration (Ohura and Kamiya, 2018a), however, the property and the underlying mechanism of after potential was not thoroughly studied experimentally, especially in thin axons in the central nervous system, because of the extremely small size of axon diameter does not allow direct recordings from the axonal membrane in thin axons.

So far, several studies tried to characterize the axonal after potential by direct recording from axon terminals exceptionally large enough for patch-clamp recording, i.e., calyx of Held (Borst et al., 1995; Kim et al., 2010) and hippocampal mossy fiber terminal (Ohura and Kamiya, 2018b). Although previous studies have suggested that after potential at soma was mediated by several different mechanisms (Raman and Bean, 1997; Yue and Yaari, 2004; Gu et al., 2005; Metz et al., 2005; Yue et al., 2005; D’Ascenzo et al., 2009; Martinello et al., 2015). In this study, it was attempted to describe the quantitative contribution of the capacitive component in axonal after potential by a numerical simulation of membrane potential. For this purpose, we adopted a realistic model of hippocampal mossy fiber (Engel and Jonas, 2005) assuming typical *en passant* structure with large boutons implemented with voltage-dependent Na^+^- and K^+^ conductance reflecting properties of those recorded from mossy fiber terminals. A series of simulation analysis revealed the relative contribution of the capacitive component due to the passive propagation of upstream action potential. The simulation also illustrated the characteristic voltage-dependency of after potential recorded from mossy fiber terminals (Ohura and Kamiya, 2018b). The early phase of after potential is mainly determined by voltage-dependent K^+^ conductance (Storm, 1987; Geiger and Jonas, 2000; Dodson et al., 2003), while the later phase is mediated by capacitive discharges of the axonal membrane. The sequential contribution of voltage-dependent K^+^ conductance and capacitive component nicely reconstructed the time course and voltage-dependency of axonal after potential observed experimentally (Geiger and Jonas, 2000; Ohura and Kamiya, 2018b).

## Materials and Methods

### Simulation

The simulated membrane potential (V_m_) was calculated according to the model suggested by Engel and Jonas (2005) based on the data recorded from mossy fiber boutons. The model basically assumed a Hodgkin Huxley-type gating model adapted to channels those recorded in mossy fiber terminals, and K^+^ channel inactivation (Geiger and Jonas, 2000) was reconstructed by implementing multiplicatively with parameters of recombinant K_V_1.4 channels (Wissmann et al., 2003). Simulations were performed using NEURON 7.5 for Windows (Hines and Carnevale, 1997). The passive electrical properties of the axon were assumed to be uniform, with a specific membrane capacitance Cm of 1 μF cm^−2^, a specific membrane resistance Rm of 10,000 Ω cm^2^, and an intracellular resistivity Ri of 110 Ω cm. The structure of the mossy fiber (Acsády et al., 1998; Henze et al., 2000) was approximated by a soma (diameter, 10 μm), 10 axonal cylinders (diameter, 0.2 μm; length, 100 μm), and 10 *en passant* boutons (diameter, 4 μm). The number of segments was 1 μm^−1^, and the time step (dt) was 5 μs in all simulations. The resting potential was assumed to be −80 mV, although it was changed to −90 or −100 mV in some series of simulation. The reversal potential of the leak conductance was set to −81 mV to maintain stability. Voltage-gated Na^+^ channels, K^+^ channels, and leakage channels were inserted into the soma, axon, and boutons, respectively. The Na^+^ conductance density was set to 50 mS cm^−2^ for the axon and boutons and 10 mS cm^−2^ for the soma. The K^+^ conductance density was set to 36 mS cm^−2^ throughout all parts of the neurons. Action potentials were evoked by injection of depolarizing current into the 9th bouton (0.2 ms, 0.1 or 0.2 nA) or the soma (2 ms, 0.2 nA). The equilibrium potentials for Na^+^ and K^+^ ions were assumed to be +50 and −85 mV, respectively. In the simulation in Figure 5, equilibrium potentials of K^+^ ions were varied from −65 to −105 mV,

## Results

### Passive Propagation of Action Potential Along the Mossy Fiber Model

**FIGURE 1:**
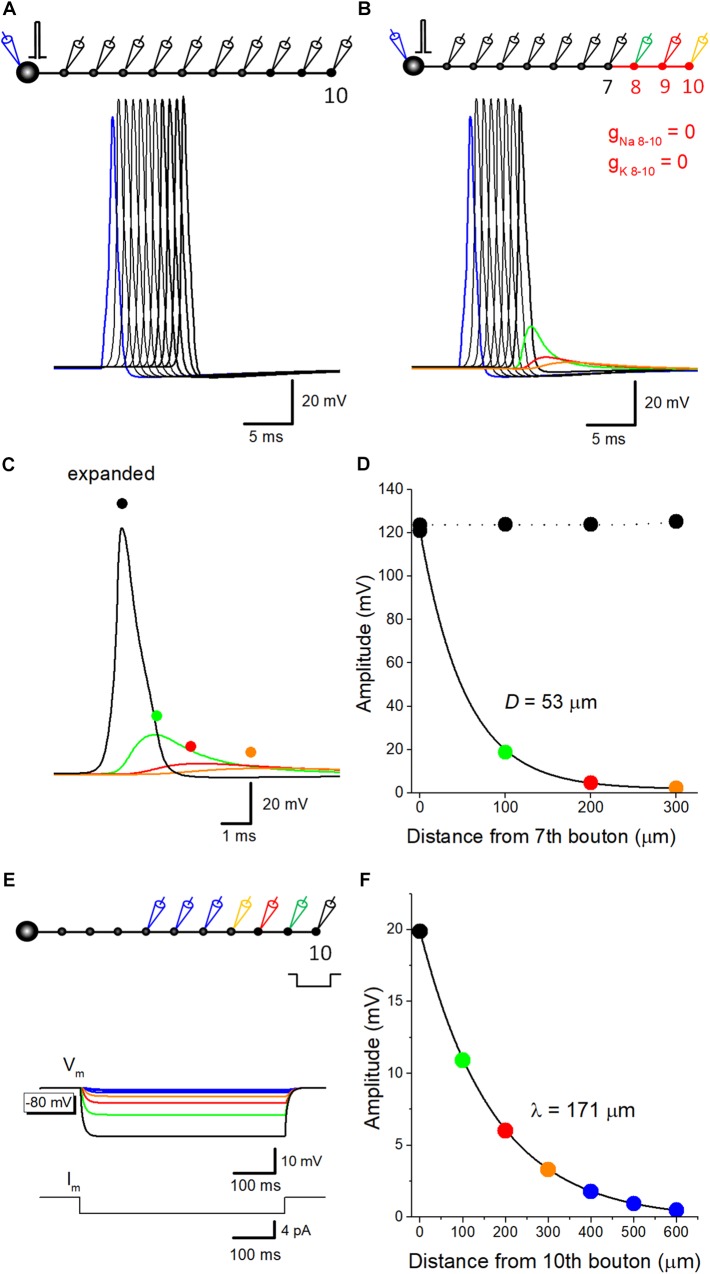
Estimation of passive propagation of action potential along a hippocampal mossy fiber model. **(A)** Reliable propagation of action potential throughout *en passant* axon with 10 boutons evenly spaced every 100 μm (“a pearl chain model”). Brief current injection into the soma elicited action potential which propagates faithfully to the 10th bouton without attenuation. **(B)** Distance-dependent decay of the amplitude of action potential without voltage-dependent ionic conductance. Voltage-gated Na^+^- and K^+^-channels were omitted from the distal portion of mossy fiber axon from 8th (*green*) to 10th (*orange*) boutons and axon, as shown in *red*. The amplitude of depolarization decreased successively, and the time course was slowed down along the distance, consistent with the notion that it reflects passive propagation due to capacitive discharge by an upstream action potential. **(C)** Traces in **B** were expanded in time for comparison. **(D)** Spatial profiles of passive propagation. The decay of the peak amplitude along the distance was fitted by a single exponential with 53 μm for the length with a reduction to 1/e (37%). The amplitude of propagating action potentials with Na^+^- and K^+^-channels (*black*) was shown for comparison. **(E)** Hyperpolarizing responses to long pulse current injection of 4 pA for 500 ms into the 10th bouton, which were used for estimation of the length constant of steady-state hyperpolarization. **(F)** The decay of the peak amplitude along the distance was fitted by a single exponential with 171 μm.

Capacitive discharge of axonal membrane due to upstream action potential might contribute to a downstream action potential. To evaluate the relative contribution of components reflecting passive propagation, voltage-dependent Na^+^- and K^+^ conductance was removed from distal portions of mossy fibers, and spatial profile of depolarization was examined. In the control condition, reliable propagation of action potential was reconstructed by simulation (Figure 1A). A small increase in the peak amplitude at the 10th bouton is possibly due to the seal end effect at the last bouton in the model. When Na^+^- and K^+^ conductance was omitted from distal axons from the eighth axon and bouton, substantial depolarization propagated passively (Figure 1B), but the amplitude declined along with the distance and the time course was slowed (Figure 1C), possibly reflecting the electrical filtering property of axon cable. The amplitude was plotted against distance from the 7th bouton, end of the compartment which expresses normal Na^+^ and K^+^ conductance. Data points were fitted with single exponential, and the distance with a reduction to 1/e (37%) was evaluated as 53 μm (Figure 1D). These results suggest that passively propagated depolarization partly contributed to the after potential recorded from downstream boutons. Conversely, passive propagation from the downstream compartment may affect upstream action potential. Passive depolarization may spatially distribute both backward and forward directions at a certain time point, although ionic component travels only forward direction by propagation of action potential. In support of this notion, action potential recorded at the 7th bouton was slightly smaller than that recorded from the upstream bouton as illustrated in Figure 1B. The rising phase of action potentials at downstream bouton concurs with the peak of an action potential at the upstream bouton, and therefore affecting the peak of the upstream action potential.

The value of spatial decay constant of 53 μm for passive propagation was relatively short. This may partly due to the filtering of fast voltage transient of the action potential by axon cable. In line with this notion, the length constant of mossy fiber model, as measure by spatial decay of steady-state hyperpolarization in response to current injection of longer step-pulse (4 pA for 500 ms, Figure 1E) was estimated as 171 μm (Figure 1F). This value is much close to that evaluated for EPreSP (subthreshold passive propagated somatic EPSP at mossy fiber axon (430 μm, Alle and Geiger, 2006).

### Voltage-Dependency of Axonal After Potential in Mossy Fiber Model

**FIGURE 2:**
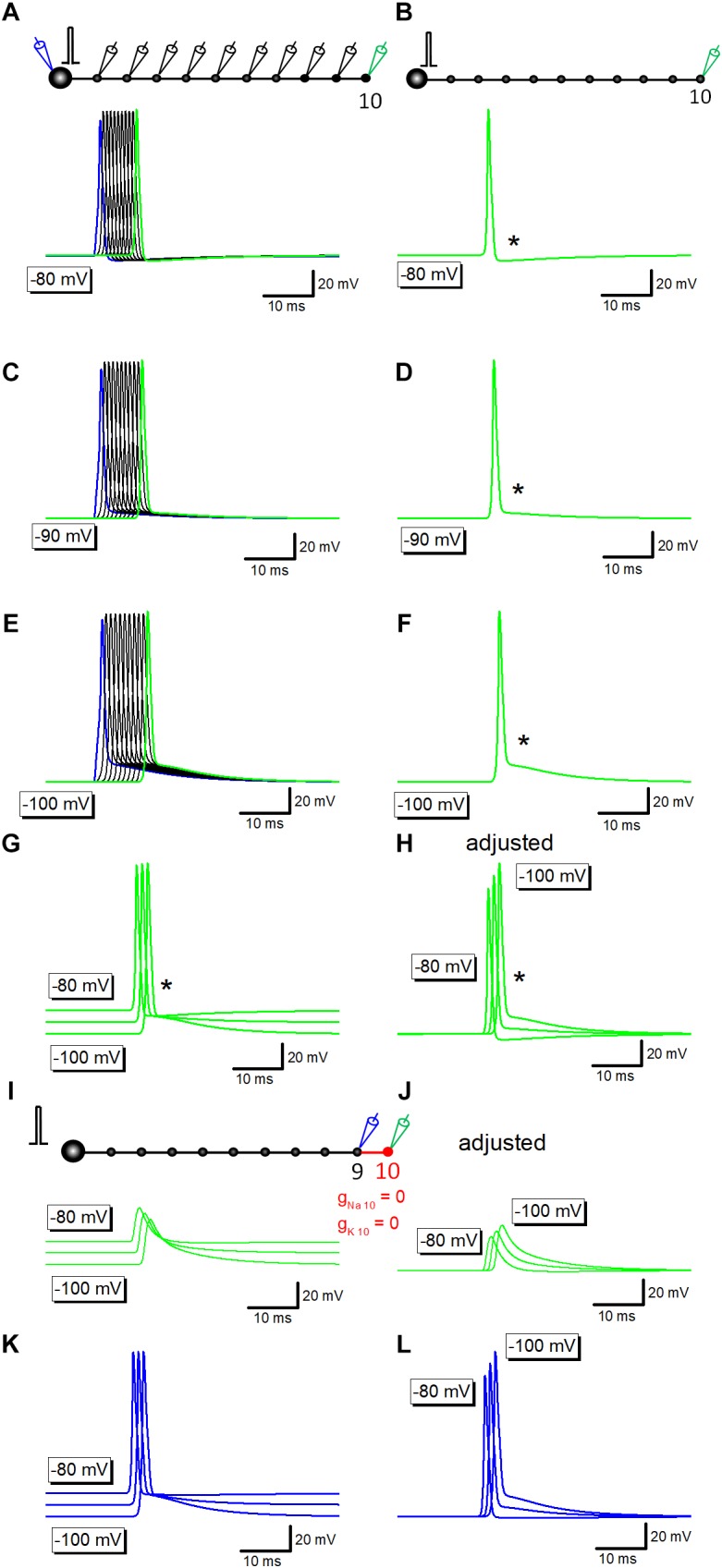
Voltage-dependency of components of axonal after potential. **(A)** Propagating action potentials recorded from every bouton in which the resting potential was set at –80 mV. **(B)** Action potential at the 10th bouton was shown in *green*. An asterisk (^∗^) shows the hyperpolarizing after potential. **(C–F)** Similar traces in which the resting potential was set at –90 mV **(C,D)** or at –100 mV **(E,F)**. After potential (^∗^) was reversed in polarity to depolarizing one. **(G)** Superimposed traces in panels **(B,D,F)**. **(H)** Traces in panel **(G)** were aligned by subtracting baseline soon before the response to compare the time course of after potential at various membrane potentials. **(I)** Capacitive component due to passive propagation was calculated by removing Na^+^- as well as K^+^-channels from 10th axon and bouton (*red*). **(J)** Traces in panel **(I)** were adjusted for the baseline to compare the time course. **(K)** Action potential waveforms at the 9th bouton in the above simulation. **(L)** Traces in panel **(K)** were adjusted for the baseline to compare the time course.

Previous studies in the calyx of Held (Sierksma and Borst, 2017), in the cerebellar basket cells (Begum et al., 2016), and in the hippocampal mossy fibers (Ohura and Kamiya, 2018b), have shown that the amplitude of after potential was negatively correlated with the initial membrane potentials. To test if a similar voltage dependency is reconstructed in simulation, resting membrane potential was changed by altering the equilibrium potential of leak conductance. At resting potential of −80 mV, after potentials are hyperpolarizing in polarity (Figure 2A,B). At −90 or −100 mV, depolarizing after potential with similar time course with those recorded experimentally were reconstructed (Figure 2C–F).

Superimposed traces of the reconstructed action potentials and after potentials (Figure 2G,H) clearly illustrated that the early phase, the initial 5 ms, converges around equilibrium potential of K^+^ ions (−85 mV) as shown by an asterisk. It should be noted that the action potential onset was delayed at −90 or −100 mV (Figure 2G,H), possibly reflecting the slightly slower conduction at these negative membrane potentials than at −80 mV.

Simulation at −80 to −100 mV in which voltage-dependent Na^+^- and K^+^ conductance was removed from distal portions of mossy fibers shown in red (Figure 2I) left capacitive component which basically shows no voltage-dependency as in Figure 2G, although the small difference in amplitude (Figure 2J) possibly reflect changes in the amplitude of upstream action potential at different membrane potentials (at −80 to −100 mV). In line with this notion, action potentials at the 9th bouton show similar changes in amplitude as in Figure 2K,L. The difference in onset of action potentials (Figure 2K,L) and the downstream capacitive components (Figure 2K,L) may also be caused by the slower conduction at −90 or −100 mV.

### Ionic and Capacitive Components Underlying Propagating Action Potential

**FIGURE 3:**
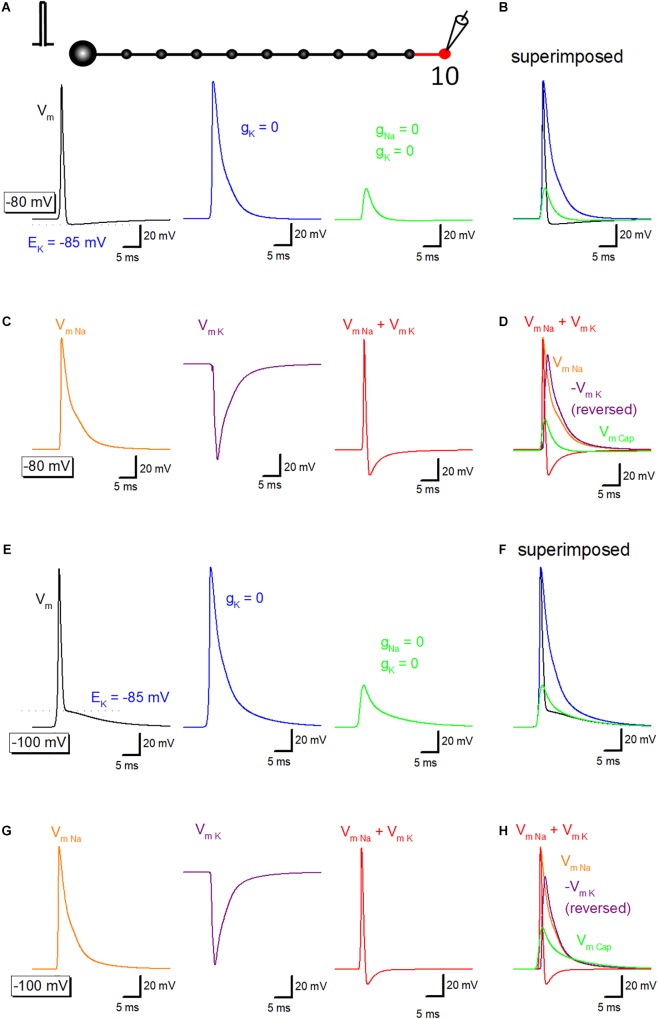
Ionic and capacitive components underlying propagating action potential. **(A)** Reconstructed action potentials at the 10th boutons in the model (left, *black*). Equilibrium potential of K^+^ ions (E_K_, –85 mV) was shown as a *blue* dotted line. When voltage-dependent K^+^ conductance was removed from 10th axon shaft and 10th bouton illustrating in the red, decay phase of the action potential was significantly delayed (middle, *blue*). Removal of voltage-dependent Na^+^ conductance as well largely reduced the amplitude (right, *green*). **(B)** Superimposed traces in panel **(A)**. **(C)** Calculated components due to activation of voltage-dependent Na^+^ channels (V_mNa_, *orange*), K^+^ channels (V_mK_, *purple*) and the sum of them (V_mNa_ + V_mK_, *red*). **(D)** Superimposed traces in **C**. **(E–H)** Similar data from panels **(A–D)** except for the resting potential was set at –100 mV. E_K_ (–85 mV) was also shown as a *blue* dotted line in Panel **(E)**.

To get insight into the relative contribution of ionic and capacitive mechanisms in shaping time course of axonal after potential, a series of simulation to replace voltage-dependent Na^+^ and K^+^ conductance in the axonal membrane were carried out. Propagating action potential recorded from the 10th bouton (Figure 3A, *black*) was broadened by removal of voltage-dependent K^+^ conductance from the 10th axon and bouton as shown in *red* (Figure 3A, *blue*). Further removal of voltage-dependent Na^+^ conductance reduced the amplitude substantially (Figure 3A, *green*), although substantial depolarization, which possibly reflects the capacitive component due to passive propagation of upstream action potential. Superimposed traces in Figure 3B shows crosspoint between *black* and *green* traces, suggesting that K^+^ conductance curtails passive components to shape the characteristic waveform of after potential. Using these data, components of membrane potential changes due to activation of Na^+^ channels (V_mNa_, *orange*) or K^+^ channels (V_mK_, *purple*) were calculated by subtraction. Sum of V_mNa_ and V_mK_ was calculated and shown in *red* (Figure 3C). Superimposed traces in Figure 3D clearly illustrate the temporal relationship of these components.

Similar simulation analysis was also performed at the resting potential of −100 mV, in which the waveform of after potential (Figure 3E,F) closely resembles with those of the recorded waveform of depolarizing after potential in the experiment (Geiger and Jonas, 2000; Ohura and Kamiya, 2018b). Na^+^- (*orange*) and K^+^-channel dependent (*purple*) components were calculated (Figure 3G), and the time course was compared with capacitive component (*green*) isolated by removing Na^+^- and K^+^- conductance. Figure 3H also illustrated that the relative contribution of each component, although a small effect of shunting of passive depolarization by large ionic components could not be excluded entirely. The amplitude of the early phase of after potential is close to the equilibrium potential of K^+^ ions (E_K_, −85 mV), in line with the notion that the early phase is dominantly determined by the K^+^ channel-dependent component.

### Capacitive Current Underlying Propagating Action Potential

**FIGURE 4:**
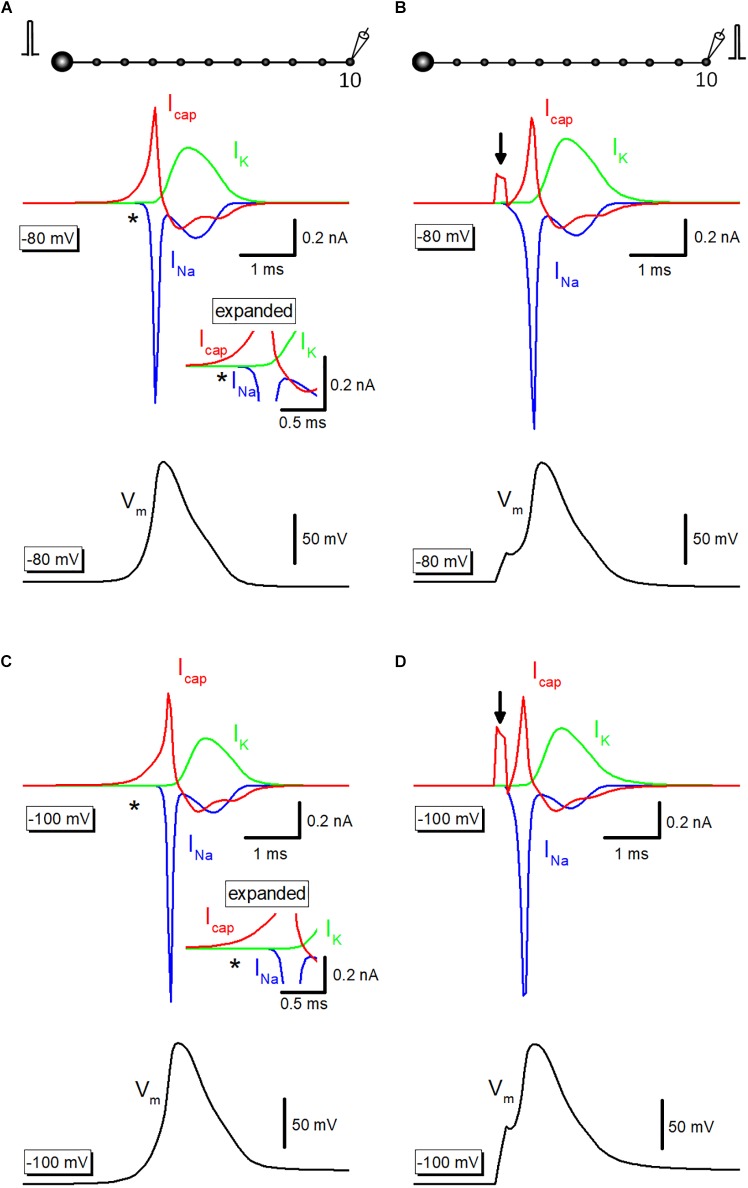
Capacitive current during propagating an action potential. **(A)** Capacitive current (I_cap_, *red*) during propagating action potential (*black*) were shown with Na^+^- (I_Na_, *blue*) and K^+^-current (I_K_, *green*). I_Na_ initiated with a delay from the onset of I_cap_ as shown by an asterisk (^∗^). The inset represents the time-expanded traces showing the delay from capacitive (I_cap_) to the ionic (I_Na_ and I_K_) components. **(B)** I_cap_ (*red*), I_Na_ (*blue*), and I_K_ (*green*) during action potential elicited by current injection into the recorded bouton (*black*). Arrow indicates capacitive current due to injected current. **(C,D)** Similar data with **A,B** except for the resting potential was set at –100 mV.

To illustrate the exact time course and the sequence of activation of ionic and capacitive components, capacitive current (I_cap_) propagating from upstream axon was simulated. Outward I_cap_ (*red*) preceded the onset of inward Na^+^-current (I_Na_, *blue*) and outward K^+^-current (I_K_, *green*) during propagating action potential elicited by somatic stimulation (Figure 4A), as highlighted with an asterisk (^∗^). The delay in activation of ionic currents reflects passive propagation due to capacitive discharge from the upstream axonal membrane. In contrast, when action potentials were evoked by current injection to the recorded bouton, there was no delay between the onsets of I_cap_ and I_Na_ (Figure 4B). Arrow indicates capacitive current due to current injection. The delay from capacitive to the ionic component is much obvious when the resting potential was set at −100 mV (Figure 4C). Direct stimulation at the recorded bouton also elicited I_cap_ and I_Na_ without delay at −100 mV (Figure 4D).

### Potassium Conductance Determines the Initial Phase of After Potential

**FIGURE 5:**
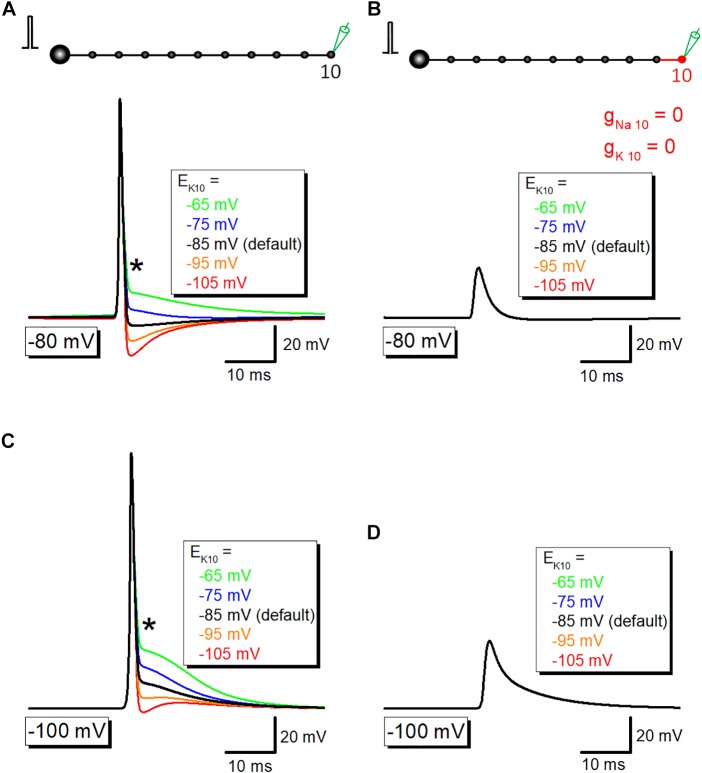
The contribution of K^+^ conductance in the initial phase of after potential. **(A)** The dependency of after potential on equilibrium potential for K^+^ ions around the 10th axon and bouton (E_K10_). The initial phase of after potential, as marked by an asterisk (^∗^), was linearly changed with alteration of E_K_. **(B)** Capacitive component of passive propagation, isolated by removal of Na^+^-and K^+^-conductance, was unchanged by alteration of E_K_. **(C,D)** Similar data with panels **(A,B)** except for the resting potential was set at –100 mV.

Then it was examined whether potassium channel-dependent fast repolarization dominate time course of the initial phase of after potential. For this purpose, the equilibrium potential for K^+^ ions around the 10th axon and bouton (E_K10_) was changed systematically to vary the driving force of K^+^ ions across the axonal membrane. Upon changes in E_K10_ from −65 to −105 mV, initial phase (or breakpoint) was changed (Figure 5A). When E_K10_ was set at more positive (−65 or −75 mV) than resting potential (−80 mV), after potential reversed polarity to depolarizing. A passive propagating component as measured by blocking voltage-dependent Na^+^- and K^+^ conductance was unchanged by an alteration in E_K10_ (Figure 5B). Similar results were obtained when resting potentials were set at −100 mV (Figure 5C,D). These results suggest that activation of K^+^ conductance determines fast repolarization and the breakpoint of the initial phase of after potential.

### The Contribution of Passive Capacitive Discharge in the Late Phase of After Potential

**FIGURE 6:**
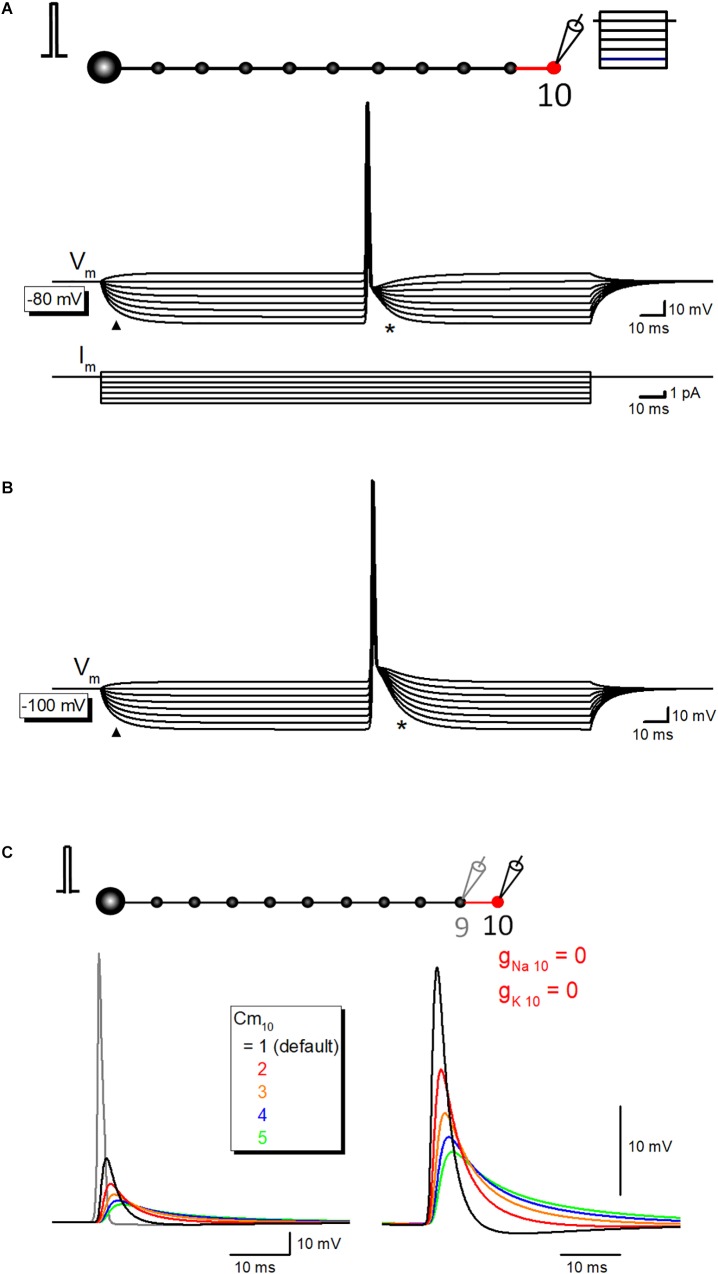
The contribution of passive capacitive discharge in the late phase of after potential. **(A)** Propagating action potential-after potential sequence recorded at various membrane potentials in response to stepwise current injection (from +1 to –6 pA, 1 pA steps) to the recorded 10th bouton whose resting membrane potential was set at –80 mV. Note that the slow relaxation of membrane potential in response to stepwise current injection (triangle) shows a similar time course with that of after potential (asterisk). Lower traces are showing step-pulse current injection. **(B)** Similar records at –100 mV initial membrane potential. Again, time courses of hyperpolarization to step current injection (triangle) are similar to that of after potential. **(C)** Effect of the changes in the specific membrane capacitance Cm on passive capacitive component measured by removal of g_Na_ and g_K_ from the 10th axon and bouton.

Time course of the slow phase of afterpotential seems to be slow enough to be explained by the passive properties of the axonal membrane. To test this notion, the simulation of propagating action potential with stepwise current injection was performed. Time course of slow relaxation of membrane potential upon current step injection (triangle) was quite similar to that of the late phase of afterpotential (asterisk) as shown in Figure 6A. The time constant of hyperpolarizing responses was 7.9 ms, while that of the late phase of afterpotential was 7.1 ms. The similarity in the time courses was also illustrated in the simulation data with the initial membrane potential at −100 mV (Figure 6B). The contribution of capacitive components was also supported by the simulation changing the value of specific membrane capacitance (Cm) systematically. As expected from cable filtering by capacitance, increase in Cm reduce and prolonged the passive propagating component as shown in Figure 6C. Taking together, it was suggested that the capacitive component of passive propagation contribute substantially to the generation of depolarizing after potential.

## Discussion

In this study, a series of numerical simulation using a realistic model of hippocampal mossy fiber was performed to illustrate the possible mechanisms underlying after potential following an axonal action potential. The early phase of after potential converges on the equilibrium potential of K^+^ ions (E_K_) and thereby the dominant contribution of voltage-dependent K^+^ channels was suggested. On the other hand, the later phase was most likely explained by the passive capacitive discharge of the axonal membrane. A characteristic waveform of action potential-after potential sequence was nicely reconstructed by a combination of a breakpoint due to fast repolarization by the opening of K^+^ channels and slow relaxation of polarization by the capacitive discharge of the axonal membrane.

### Evaluation of Passive Propagation of Action Potential Along the Mossy Fiber Model

In an experimental approach, it is difficult to estimate the spatial and temporal distribution of a passive component in a propagating action potential along the axon. Recordings from at least two distinct sites of the connecting axon, as well as spatially restricted blocking of voltage-gated conductance to eliminate an active component, are necessary for the evaluation of passive properties of axonal membranes (de San Martin et al., 2017). These requirements are difficult to be achieved in the experimental conditions, while simulation readily figures out passive components in a propagating action potential.

The amplitude of passive components reflecting action potential at the next *en passant* bouton is estimated as 19 mV. The simulation also showed that the amplitude of the passive component declined along with the distance. The decay of the amplitude of passive components was fit with single exponential with the length for a reduction to 1/e as 53 μm. This value was similar to that estimated for GABA_A_-receptor mediated response in axons of cerebellar molecular layer interneurons and in axons of cultured Purkinje cells (de San Martin et al., 2015, 2017). Passive component inevitably involves in a substantial fraction in propagating action potential-after potential sequence. This notion should be directly tested experimentally by subcellular recording approach from two distinct sites of a connecting mossy fiber axon.

The amplitudes of experimentally observed depolarizing after potentials at mossy fiber terminals are 6.9 ± 0.6 mV in the rat (Geiger and Jonas, 2000) and 15.3 ± 1.3 mV in the mouse (Ohura and Kamiya, 2018b). In our simulation, the action potential of 121 mV was decayed to 19 mV at 100 μm and 5 mV at 200 μm by passive propagation as shown in Figure 1D, suggesting that inter-bouton distances are ranged between 100 and 200 μm. Although the cable properties also depend on Cm value and the diameter of mossy fibers, the simple model suggested by Engel and Jonas (2005), which we adopted in this study, reasonably approximated the experimental values of after potential amplitude.

### Voltage-Dependent K^+^ Channels Provide a Breakpoint in the Early Phase of After Potential

Previous studies consistently suggested that axonal after potential was mediated by the capacitive discharge of the axonal membrane. Contrary to expected from passive nature of capacitive component, however, it was shown that the amplitude of after potential shows clear voltage-dependency, i.e., after potential was smaller at depolarizing membrane potential, while it got bigger at hyperpolarizing membrane potential (Begum et al., 2016; Sierksma and Borst, 2017; Ohura and Kamiya, 2018b). The results of simulation in this study revealed that the characteristic waveform of after potential was reconstructed by a combination of fast repolarization by voltage-gated K^+^-channels and by the passive capacitive discharge of the axonal membrane. Time course of the late phase of after potential was similar to those of slow relaxation of membrane potential in response to stepwise hyperpolarizing current injection, in consistent with the passive nature of the capacitive component.

Combination of voltage-dependent K^+^ conductance and capacitive discharge might be a too simplified interpretation of the characteristic time course of after potential. Since previous studies pointed out that other voltage-dependent conductance, such as resurgent (Kim et al., 2010; Ohura and Kamiya, 2018b) as well as persistent Na^+^ current (Yue et al., 2005), are involved in the late phase of after potential. These voltage-dependent currents might additionally involve in boosting up the after potential. Several previous studies reported that after potential waveform was altered upon changes in the recording membrane potentials, and sometimes reverse in polarity at depolarized membrane potentials. The reversal potentials estimated or extrapolated from the recorded after potential waveforms varies considerably (Begum et al., 2016; Sierksma and Borst, 2017; Ohura and Kamiya, 2018b), possibly reflecting the various contribution of active (voltage-dependent conductance) and passive (capacitive discharge) components. Overall voltage-dependency is consistent with the notion that early phase is predominantly governed by voltage-dependent K^+^ conductance since the initial phase seems to reverse around E_K_. The later phase is likely to reflect voltage-independent passive components of slow depolarization. These notions suggested that voltage-dependent K^+^ channels and capacitive discharge involve as common mechanisms to shape the framework of after potential, while active conductance such as resurgent Na^+^ current or T-type Ca^2+^ current may additively contribute to the late phase of after potential.

One may argue that the model of hippocampal mossy fiber which we adopted in this study (Engel and Jonas, 2005) is too simple. For instance, if voltage-dependent conductance with activation voltage near resting potential like Ih or the persistent-type Na^+^ channels is expressed in mossy fibers, depolarizing after potential might be affected by these voltage-dependent conductance. So far, there is no evidence for the presence of Ih on mossy fibers (Chevaleyre and Castillo, 2002; but see Mellor et al., 2002). Presence of persistent Na^+^ channels on mossy fibers is also less likely since Na^+^ current recorded from mossy fiber fully inactivate during prolonged depolarization step (Engel and Jonas, 2005).

Passive depolarization might be shunted by large ionic conductance increase during an action potential and thereby reduced passive depolarization to some extent. It should be mentioned, as demonstrated in Figure 4A,C, capacitive current (Icap) often precede I_Na_ during an action potential, while still some fraction overlapped in the time domain. This implies shunting of passive depolarization, if any, is limited to the falling phase of Icap.

### Possible Contribution of Slow Activating Voltage-Dependent Na^+^ Current in After Potential

Mossy fiber model adopted in this study assumes equilibrium potentials of K^+^ ions (E_K_) at −85 mV. In this model, action potentials were followed by hyperpolarizing after potential at resting potential of −80 mV. This is expected if the initial phase of after potentials is governed by the opening of voltage-gated K^+^ channels and thereby approaching to E_K_ more negative than resting membrane potential. However, experimentally recorded action potentials from mossy fiber terminals displays robust depolarizing after potential (Geiger and Jonas, 2000; Ohura and Kamiya, 2018b), and the waveforms are quite similar to those obtained in the simulation assuming more positive E_K_ value than the resting potential of −80 mV. The reason for the difference in polarity between experiment (depolarizing) and the model (hyperpolarizing) needs to be clarified in future studies. It seems to be reasonable to assume additional involvement of slow activating voltage-dependent Na^+^ current such as resurgent Na^+^ current (Ohura and Kamiya, 2018b) in boosting slow depolarization observed in the experiment.

In summary, systematic simulation analysis using a model of mossy fibers revealed that action potential propagates passively via axon cable substantially, and thereby consists of a part of after potential following an action potential. The characteristic waveform of axonal action potential-after potential sequence, as well as apparent voltage-dependency of after potential, are reconstructed with the simple combination voltage-dependent K^+^ channel component and a passive capacitive component. An initial breakpoint after action potential may be controlled by fast repolarization by the opening of K^+^ channels, while the later phase is dominantly mediated by the capacitive discharge of the axonal membrane. These notions may shed light on the common underlying mechanism of axonal after potential, although specific mechanisms may involve in fine-tuning of the axonal excitability.

## Data Availability

The raw data supporting the conclusions of this manuscript will be made available by the authors, without undue reservation, to any qualified researcher.

## Author Contributions

HK performed the simulation, analyzed the data, and wrote the manuscript.

## Conflict of Interest Statement

The author declares that the research was conducted in the absence of any commercial or financial relationships that could be construed as a potential conflict of interest.

## References

[B1] Acsády L. Kamondi A. Sík A. Freund T. Buzsáki G. (1998). GABAergic cells are the major postsynaptic targets of mossy fibers in the rat hippocampus. *J. Neurosci.* 18 3386–3403. 10.1523/jneurosci.18-09-03386.1998 9547246 PMC6792657

[B2] Alle H. Geiger J. R. P. (2006). Combined analog and action potential coding in hippocampal mossy fibers. *Science* 311 1290–1293. 10.1126/science.1119055 16513983

[B3] Barrett E. F. Barrett J. N. (1982). Intracellular recording from vertebrate myelinated axons: mechanism of the depolarizing afterpotential. *J. Physiol.* 323 117–144. 10.1113/jphysiol.1982.sp014064 6980272 PMC1250348

[B4] Bean B. P. (2007). The action potential in mammalian central neurons. *Nat. Rev. Neurosci.* 8 451–465. 10.1038/nrn2148 17514198

[B5] Begum R. Bakiri Y. Volynski K. E. Kullmann D. M. (2016). Action potential broadening in a presynaptic channelopathy. *Nat. Commun.* 7:12102 10.1038/ncomms12102 PMC4935806 27381274

[B6] Borst J. G. Helmchen F. Sakmann B. (1995). Pre- and postsynaptic whole-cell recordings in the medial nucleus of the trapezoid body of the rat. *J. Physiol.* 489 825–840. 10.1113/jphysiol.1995.sp021095 8788946 PMC1156851

[B7] Chevaleyre V. Castillo P. E. (2002). Assessing the role of Ih channels in synaptic transmission and mossy fiber LTP. *Proc. Natl. Acad. Sci. U.S.A.* 99 9538–9543. 10.1073/pnas.142213199 12093909 PMC123176

[B8] D’Ascenzo M. Podda M. V. Fellin T. Azzena G. B. Haydon P. Grassi C. (2009). Activation of mGluR5 induces spike afterdepolarization and enhanced excitability in medium spiny neurons of the nucleus accumbens by modulating persistent Na^+^ currents. *J. Physiol.* 587 3233–3250. 10.1113/jphysiol.2009.172593 19433572 PMC2727034

[B9] David G. Modney B. Scappaticci K. A. Barrett J. N. Barrett E. F. (1995). Electrical and morphological factors influencing the depolarizing after-potential in rat and lizard myelinated axons. *J. Physiol.* 489 141–157. 10.1113/jphysiol.1995.sp021037 8583398 PMC1156799

[B10] de San Martin J. Z. Jalil A. Trigo F. F. (2015). Impact of single-site axonal GABAergic synaptic events on cerebellar interneuron activity. *J. Gen. Physiol.* 146 477–493. 10.1085/jgp.201511506 26621773 PMC4664828

[B11] de San Martin J. Z. Trigo F. F. Kawaguchi S. Y. (2017). Axonal GABA_A_ receptors depolarize presynaptic terminals and facilitate transmitter release in cerebellar Purkinje cells. *J. Physiol.* 595 7477–7493. 10.1113/JP275369 29072780 PMC5730858

[B12] Debanne D. Campanac E. Bialowas A. Carlier E. Alcaraz G. (2011). Axon physiology. *Physiol. Rev.* 91 555–602. 10.1152/physrev.00048.2009 21527732

[B13] Dodson P. D. Billups B. Rusznák Z. Szûcs G. Barker M. C. Forsythe I. D. (2003). Presynaptic rat Kv1.2 channels suppress synaptic terminal hyperexcitability following action potential invasion. *J. Physiol.* 550 27–33. 10.1113/jphysiol.2003.046250 12777451 PMC2343026

[B14] Engel D. Jonas P. (2005). Presynaptic action potential amplification by voltage-gated Na^+^ channels in hippocampal mossy fiber boutons. *Neuron* 45 405–417. 10.1016/j.neuron.2004.12.048 15694327

[B15] Gardner-Medwin A. R. (1972). An extreme supernormal period in cerebellar parallel fibres. *J. Physiol.* 222 357–371. 10.1113/jphysiol.1972.sp009802 5033469 PMC1331386

[B16] Geiger J. R. Jonas P. (2000). Dynamic control of presynaptic Ca^2+^ inflow by fast-inactivating K^+^ channels in hippocampal mossy fiber boutons. *Neuron* 28 927–939. 10.1016/s0896-6273(00)00164-1 11163277

[B17] Gu N. Vervaeke K. Hu H. Storm J. F. (2005). Kv7/KCNQ/M and HCN/h, but not K_Ca_2/SK channels, contribute to the somatic medium after-hyperpolarization and excitability control in CA1 hippocampal pyramidal cells. *J. Physiol.* 566 689–715. 10.1113/jphysiol.2005.086835 15890705 PMC1464792

[B18] Henze D. A. Urban N. N. Barrionuevo G. (2000). The multifarious hippocampal mossy fiber pathway: a review. *Neuroscience* 98 407–427. 10.1016/s0306-4522(00)00146-9 10869836

[B19] Hines M. L. Carnevale N. T. (1997). The NEURON simulation environment. *Neural. Comput.* 9 1179–1209. 10.1162/neco.1997.9.6.1179 9248061

[B20] Kamiya H. Ozawa S. Manabe T. (2002). Kainate receptor-dependent short-term plasticity of presynaptic Ca^2+^ influx at the hippocampal mossy fiber synapses. *J. Neurosci.* 22 9237–9243. 10.1523/jneurosci.22-21-09237.2002 12417649 PMC6758040

[B21] Kim J. H. Kushmerick C. von Gersdorff H. (2010). Presynaptic resurgent Na^+^ currents sculpt the action potential waveform and increase firing reliability at a CNS nerve terminal. *J. Neurosci.* 30 15479–15490. 10.1523/JNEUROSCI.3982-10.2010 21084604 PMC3073539

[B22] Malenka R. C. Kocsis J. D. Ransom B. R. Waxman S. G. (1981). Modulation of parallel fiber excitability by postsynaptically mediated changes in extracellular potassium. *Science* 214 339–341. 10.1126/science.7280695 7280695

[B23] Martinello K. Huang Z. Lujan R. Tran B. Watanabe M. Cooper E. C. (2015). Cholinergic afferent stimulation induces axonal function plasticity in adult hippocampal granule cells. *Neuron* 85 346–363. 10.1016/j.neuron.2014.12.030 25578363 PMC4306544

[B24] Meeks J. P. Mennerick S. (2004). Selective effects of potassium elevations on glutamate signaling and action potential conduction in hippocampus. *J. Neurosci.* 24 197–206. 10.1523/jneurosci.4845-03.2004 14715952 PMC6729587

[B25] Mellor J. Nicoll R. A. Schmitz D. (2002). Mediation of hippocampal mossy fiber long-term potentiation by presynaptic Ih channels. *Science* 295 143–147. 10.1126/science.1064285 11778053

[B26] Metz A. E. Jarsky T. Martina M. Spruston N. (2005). R-type calcium channels contribute to afterdepolarization and bursting in hippocampal CA1 pyramidal neurons. *J. Neurosci.* 25 5763–5773. 10.1523/jneurosci.0624-05.2005 15958743 PMC6724888

[B27] Ohura S. Kamiya H. (2016). Excitability tuning of axons in the central nervous system. *J. Physiol. Sci.* 66 189–196. 10.1007/s12576-015-0415-2 26493201 PMC10717993

[B28] Ohura S. Kamiya H. (2018a). Short-term depression of axonal spikes at the mouse hippocampal mossy fibers and sodium channel-dependent modulation. *eNeuro* 5:ENEURO0415-17.2018. 10.1523/ENEURO.0415-17.2018 PMC5820996 29468192

[B29] Ohura S. Kamiya H. (2018b). Sodium channel-dependent and -independent mechanisms underlying axonal afterdepolarization at mouse hippocampal mossy fibers. *eNeuro* 5:ENEURO0254-18.2018. 10.1523/ENEURO.0254-18.2018 PMC6140107 30225345

[B30] Raman I. M. Bean B. P. (1997). Resurgent sodium current and action potential formation in dissociated cerebellar Purkinje neurons. *J. Neurosci.* 17 4517–4526. 10.1523/jneurosci.17-12-04517.1997 9169512 PMC6573347

[B31] Sierksma M. C. Borst J. G. G. (2017). Resistance to action potential depression of a rat axon terminal in vivo. *Proc. Natl. Acad. Sci. U.S.A.* 114 4249–4254. 10.1073/pnas.1619433114 28373550 PMC5402426

[B32] Storm J. F. (1987). Action potential repolarization and a fast after-hyperpolarization in rat hippocampal pyramidal cells. *J. Physiol.* 385 733–759. 10.1113/jphysiol.1987.sp016517 2443676 PMC1192370

[B33] Wissmann R. Bildl W. Oliver D. Beyermann M. Kalbitzer H. R. Bentrop D. (2003). Solution structure and function of the “tandem inactivation domain” of the neuronal A-type potassium channel Kv1.4. *J. Biol. Chem.* 278 16142–16150. 10.1074/jbc.m210191200 12590144

[B34] Yue C. Remy S. Su H. Beck H. Yaari Y. (2005). Proximal persistent Na^+^ channels drive spike afterdepolarizations and associated bursting in adult CA1 pyramidal cells. *J. Neurosci.* 25 9704–9720. 10.1523/jneurosci.1621-05.2005 16237175 PMC6725731

[B35] Yue C. Yaari Y. (2004). KCNQ/M channels control spike afterdepolarization and burst generation in hippocampal neurons. *J. Neurosci.* 24 4614–4624. 10.1523/jneurosci.0765-04.2004 15140933 PMC6729392

[B36] Zucker R. S. (1974). Excitability changes in crayfish motor neurone terminals. *J. Physiol.* 241 111–126. 10.1113/jphysiol.1974.sp010643 4371618 PMC1331075

